# Therapeutic potential of growth hormone-releasing hormone analogues in cardiovascular and cerebrovascular diseases: mechanisms and preclinical evidence

**DOI:** 10.3389/fphar.2026.1847309

**Published:** 2026-05-28

**Authors:** Hao-Lin Ren, Yuliang Zhang, Kefan Yang, Lu Zhang, Yueyang Liu, Ming-Sheng Zhou

**Affiliations:** 1 Department of Radiology, 1st Affiliated Hospital, Nanjing Medical University, Nanjing, China; 2 Shenyang Key Laboratory of Vascular Biology, Institute of Life Science, Shenyang Medical College, Shenyang, China

**Keywords:** analogue, cardiovascular effects, GHRH, neural protection, preclinical study

## Abstract

Growth hormone-releasing hormone (GHRH) is a hypothalamic neuropeptide that stimulates growth hormone (GH) secretion from the pituitary gland, forming the central GHRH-GH-insulin-like growth factor-1 (IGF-1) endocrine axis. Beyond its classical endocrine function, GHRH and its receptors are widely expressed in extrapituitary tissues, where they regulate diverse physiological processes including cardiovascular function and neuroprotection. Preclinical studies demonstrate that synthetic GHRH analogues like MR-409 mitigate vascular calcification in diabetes, enhance cardiac repair post-myocardial infarction, and restore diastolic function in heart failure through calcium-handling modulation. In ischemic stroke models, MR-409 exhibits neuroprotective effects, reducing mortality and promoting neural regeneration. Studies performed in animal models have demonstrated the efficacy and therapeutic benefits of these compounds in diverse cardiomyopathies and ischemic stroke. Notably, these benefits occur independently of GH/IGF-1 signaling, highlighting their therapeutic potential for cardiovascular and cerebrovascular diseases. This narrative review synthesizes evidence from animal models, underscoring GHRH analogues as promising candidates for clinical translation. The articles included were identified through systematic searches in PubMed using keywords such as “GHRH analogues”, “cardiovascular disease', and “ischemic stroke”, with selection based on relevance to therapeutic applications and preclinical efficacy.

## 1 Introduction

Cardiovascular and cerebrovascular diseases remain the leading cause of global mortality (Roth et al., 2020; Wu et al., 2026), current therapies still face critical limitations, they can alleviate symptoms in some patients, but they struggle to halt disease progression or reverse underlying pathological changes like cardiac and vascular remodeling (Hahn et al., 2024; Xing and Lin, 2025). Many patients exhibit poor responses to existing drugs, while interventions like stents or thrombectomy have narrow therapeutic windows and high recurrence rates (Gaudino et al., 2023). The rising prevalence of metabolic disorders and aging populations further exacerbates disease burden (Zile et al., 2025). Additionally, neuroprotective strategies for stroke show limited clinical efficacy despite promising preclinical data (McGrath et al., 2014). There is an urgent need for innovative approaches targeting disease-specific pathways and interventions that promote vascular regeneration rather than just symptom management.

Growth hormone-releasing hormone (GHRH) is a 44-amino acid neuropeptide primarily synthesized in the hypothalamic arcuate nucleus, GHRH represents a crucial regulator in the secretin-glucagon peptide family with remarkable structural conservation across mammalian species (Spiess et al., 1982; Ling et al., 1984). The N-terminal 1–29 amino acid sequence constitutes the bioactive core responsible for receptor binding (Frohman et al., 1986), while the C-terminal region governs molecular stability and biological potency (Rivier et al., 1982; Spiess et al., 1982). GHRH exerts its classical endocrine function through binding to GHRH receptors (GHRHR) in the anterior pituitary, stimulating growth hormone (GH) synthesis and secretion via the hypothalamus-pituitary-GH axis (Guillemin et al., 1982; Barinaga et al., 1983). However, emerging evidence reveals widespread GHRHR expression in extra-pituitary tissues, including cardiomyocytes, vascular endothelial cells, smooth muscle cells, and various neuronal populations, neural stem cells (NSCs) (Kanashiro-Takeuchi et al., 2010; Shen et al., 2018; Zhou et al., 2020; Liu et al., 2021), suggesting broader physiological roles beyond endocrine regulation. The hormone’s rapid degradation by dipeptidyl peptidase-IV (DPP-IV) (Frohman et al., 1986), which cleaves GHRH into inactive GHRH(3–44)-NH2 metabolites, has prompted extensive structural modifications to develop stable therapeutic analogues with enhanced pharmacokinetic profiles (Cai et al., 2014).

GHRH mediates its biological effects through GHRHR, a G protein-coupled receptor that primarily activates two key signaling cascades: the cyclic adenosine monophosphate (cAMP)/protein kinase A (PKA) pathway and the phosphoinositide 3-kinase (PI3K)/Akt-mitogen-activated protein kinase (MAPK) axis (Halmos et al., 2023). The cAMP/PKA pathway not only regulates GH secretion but also exerts potent anti-apoptotic effects in cardiovascular and neural tissues (Schally et al., 2019). Concurrently, PI3K/Akt signaling promotes cell survival, angiogenesis, and endothelial nitric oxide production, while MAPK/extracellular signal-regulated kinase (ERK) pathways drive cellular proliferation and tissue repair mechanisms (Wang et al., 2026). These molecular pathways collectively contribute to GHRH’s ability to mitigate oxidative stress, reduce ischemic injury, and enhance endothelial function in both cardiovascular and cerebrovascular systems (Liu et al., 2021; Ren et al., 2023). Importantly, these protective effects operate independently of the GH/insulin-like growth factor-1 (IGF-1) axis.

The therapeutic potential of GHRH in cardiovascular and cerebrovascular diseases has been significantly advanced through the development of synthetic analogues (Izdebski et al., 1995; Cai et al., 2014). Pioneering work by Nobel laureate Andrew V. Schally’s team has yielded over 100 optimized compounds, including the JI-series (e.g., JI-34) with enhanced receptor affinity and MR-series (e.g., MR-409, MR-502) analogues exhibiting prolonged half-lives and improved tissue specificity (Cai et al., 2014; Schally et al., 2019). These engineered peptides demonstrate remarkable efficacy in preclinical models of heart failure (HF), myocardial infarction (MI), stroke, and vascular dementia through multifaceted mechanisms including anti-inflammatory, anti-apoptotic, and pro-angiogenic actions (Bagno et al., 2015; Liu et al., 2021). Notably, certain GHRH antagonists like MIA-602 and MIA-690 have shown paradoxical cardioprotective effects alongside their established antitumor properties (Kanashiro-Takeuchi et al., 2012; Ma et al., 2016; Munoz-Moreno et al., 2024). The expanding repertoire of GHRH analogues, with their diverse pharmacologic profiles and tissue-specific actions, offers promising therapeutic avenues for addressing the unmet clinical needs in cardiovascular and cerebrovascular diseases, potentially revolutionizing treatment paradigms beyond conventional approaches.

This is a narrative review summarizing the therapeutic potential of GHRH and its analogues in cardiovascular and cerebrovascular diseases based on preclinical evidence. To identify relevant literature, we searched PubMed databases up to [April 2026] words including “GHRH analogues”, “cardiovascular disease”, “myocardial infarction”, “heart failure”, “ischemic stroke”, “neuroprotection”, and “angiogenesis”. Additional articles were identified from reference lists of retrieved papers. Only peer-reviewed studies published in English that focused on the therapeutic applications, mechanisms of action, or preclinical efficacy of GHRH and its analogues in cardiovascular or cerebrovascular contexts were included.

## 2 The multifaceted roles of GHRH signaling: from pituitary GH regulation to extrapituitary therapeutic applications

The hypothalamic-pituitary GHRH/GH/IGF-1 axis represents a crucial endocrine regulatory system that orchestrates physical development and metabolic homeostasis. GHRH, synthesized in the hypothalamus and transported to the anterior pituitary, binds to its cognate GHRHR on somatotrophs to stimulate GH synthesis and secretion (Barinaga et al., 1983; Halmos et al., 2023). The released GH then exerts its biological effects through both direct interaction with GH receptors distributed across various tissues and indirect mechanisms mediated by IGF-1, predominantly produced by the liver (Halmos et al., 2023). This axis regulates fundamental physiological processes including cellular proliferation, differentiation, and maturation in multiple organ systems such as bone, cartilage, skeletal muscle, and cardiovascular tissues (Zhang et al., 2020). The system maintains tight feedback regulation through both IGF-1-mediated long loop and GH-mediated short-loop mechanisms (Gusmao et al., 2025; Liu et al., 2025), ensuring proper glucose and lipid metabolism, tissue growth, and wound healing. Importantly, the discovery of functional GHRHR expression in extrapituitary tissues has revealed that GHRH signaling extends far beyond its classical endocrine role, opening new therapeutic possibilities for targeting this pathway in various diseases (Granata et al., 2025).

**FIGURE 1:**
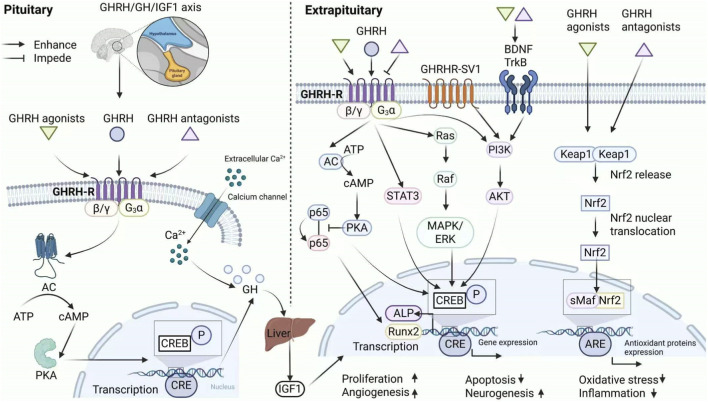
Schematic representation of the potential signaling mechanisms, cascades, and cellular effects of growth hormone-releasing hormone (GHRH), GHRH agonists, and GHRH antagonists in pituitary and extrapituitary tissues. In the anterior pituitary, GHRH binds to the GHRH receptor (GHRHR), a G protein-coupled receptor primarily coupled to Gαs. This interaction stimulates adenylate cyclase, leading the activation of the cyclic adenosine monophosphate (cAMP)/protein kinase A (PKA)/cAMP response element-binding protein (CREB) signaling pathway, which ultimately promotes GH synthesis and secretion. GH, in turn, induces the synthesis and release of insulin-like growth factor 1 (IGF-1) in the liver, forming the GHRH/GH/IGF-1 endocrine axis. Both GH and IGF-1 play critical roles in promoting cell proliferation and organ development. GHRHR and its splice variant, SV1, are widely expressed in extrapituitary tissues. Activation of GHRHR by GHRH or GHRH agonists triggers a cascade of extrapituitary biological effects, including enhanced cell proliferation, neurogenesis, and angiogenesis, as well as inhibition of apoptosis, oxidative stress, and inflammation. These effects are mediated through key signaling pathways such as mitogen-activated protein kinase (MAPK)/extracellular signal-regulated kinase 1/2 (ERK1/2), phosphatidylinositol 3-kinase (PI3K)/Akt, and Keap1/Nrf2. Conversely, blockade of GHRHR by GHRH antagonists produces opposing effects.

The GHRHR belongs to the class B family of G protein-coupled receptors (GPCR) and primarily activates the adenylate cyclase (AC)/cAMP/PKA signaling cascade (Granata et al., 2025). Upon GHRH binding, GHRHR engages Gαs proteins, stimulating cAMP production, which in turn promotes calcium influx through voltage-gated channels and GH vesicle exocytosis. The cAMP/PKA pathway further phosphorylates the transcription factor cAMP response element-binding protein (CREB), enhancing the expression of both GH and GHRHR genes via cAMP response elements (CRE) in their promoter regions (Halmos et al., 2023; Halmos et al., 2025). Additionally, GHRH activates the phospholipase C (PLC) pathway through Gβγ subunits, generating inositol triphosphate (IP3) and diacylglycerol (DAG) to mobilize intracellular calcium stores and amplify GH secretion (Halmos et al., 2025). Beyond the pituitary, GHRH signaling intersects with other critical pathways, including PI3K/Akt, ERK1/2 MAPK, and Janus kinase/signal transducer and activator of transcription (JAK/STAT), which regulate cell survival, proliferation, and metabolic functions in various tissues (Bagno et al., 2015; Zhang et al., 2020; Liu et al., 2021). Experimental studies demonstrate that GHRH protects cardiomyocytes against ischemic injury through these pathways, as inhibition of PKA, PI3K, ERK1/2, or STAT3 abolishes its cardioprotective effects (Halmos et al., 2025) (Figure 1).

While GHRHR is predominantly expressed in the anterior pituitary, it is also present in extra-pituitary tissues, including the myocardium, vascular endothelial cells, smooth muscle cells (Wang et al., 2020), mesenchymal stem cells (MSCs) (Xia et al., 2016), neural cells, and pancreas (Ludwig et al., 2010) and wide range of tumor cells (Busto et al., 2002), where it mediates diverse physiological and pathological processes. In non-pituitary tissues, GHRH signaling may involve splice variants of GHRHR, such as splice variant 1 (SV1), which lacks transmembrane domains but retains functional activity in certain cancers (Busto et al., 2002). Unlike the pituitary, where GHRH acts via classical cAMP/PKA signaling, extra-pituitary effects may involve alternative pathways, such as nitric oxide synthase (NOS)/NO/cyclic guanosine monophosphate (cGMP) when GHRHR couples with Gαs-type G proteins (Dulce et al., 2025). Additionally, GHRH exhibits autocrine/paracrine functions in tumors, where it promotes cell proliferation via MAPK and PI3K/Akt activation. In the cardiovascular system, GHRH reduces myocardial infarction size, attenuates hypertrophy, and enhances endothelial function through ERK1/2 and PI3K/Akt-dependent mechanisms. The presence of GHRHR in various cancers has led to the development of GHRH agonists (e.g., MR-409, JI-38) for tissue repair and antagonists (e.g., MIA-602, JV-1-38) for cancer therapy, highlighting its dual role in growth promotion and inhibition depending on context (Granata et al., 2025; Halmos et al., 2025).

Given its pleiotropic effects, GHRH signaling represents a promising therapeutic target for metabolic, cardiovascular and neoplastic diseases. GHRH agonists enhance pancreatic β-cell function (Zhang et al., 2015), improve cardiac repair (Bagno et al., 2015), and accelerate wound healing (Dioufa et al., 2010), making them potential treatments for diabetes and heart failure. Conversely, GHRH antagonists suppress tumor growth by blocking autocrine GHRH signaling and inhibiting the GH/IGF-1 axis, showing efficacy in prostate, breast, and renal cancers. Recent studies also suggest that GHRH antagonists (e.g., MIA-690) may synergize with chemotherapy in malignant pleural mesothelioma by impairing mitochondrial function and increasing oxidative stress (Villanova et al., 2019). Furthermore, splice variants like SV1 serve as oncogenic promoters in esophageal and endometrial cancers (Busto et al., 2002), offering new targets for precision therapy. Despite these advances, challenges remain, including the need for non-peptide small-molecule modulators and a deeper understanding of GHRH-GHRHR structural interactions. Overall, the multifaceted roles of GHRH signaling underscore its potential in both endocrine and non-endocrine therapeutic applications.

## 3 The cardiovascular effects of endogenous GHRH and GHRH analogues

**FIGURE 2:**
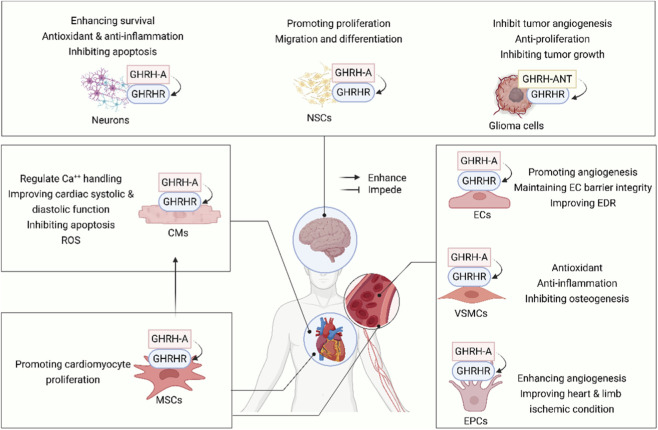
The expression of GHRH receptors and their role in mediating biological effects in the cardiovascular and central neural system. Beyond their classical pituitary expression, GHRH receptors exhibit widespread distribution in both cardiovascular and central nervous systems, being present in neural stem cells (NSCs), differentiated neurons, glioma cells, cardiomyocytes (CMs), vascular endothelial cells (ECs), and smooth muscle cells (VSMCs). In the central nervous system, GHRH agonists exert neuroprotective and regenerative effects by stimulating neural stem cell proliferation, promoting neuronal regeneration, and enhancing post-ischemic stroke recovery, while antagonists demonstrate antitumor activity through inhibition of glioma proliferation and cancer growth. Within the cardiovascular system, GHRH receptor activation mediates cardioprotection via myocardial effects (exerting anti-inflammatory, antioxidant, and anti-apoptotic actions in cardiomyocytes) and vascular effects (promoting angiogenesis, improving endothelial function, and maintaining endothelial barrier integrity). EPCs: endothelial progenitor cells; MSCs: mesenchymal stem cells.

GHRH and its synthetic analogues play a pivotal role in cardiovascular regulation through angiogenesis modulation, improvement of endothelial function, anti-inflammatory, antioxidant, and anti-apoptotic mechanisms (Shen et al., 2018; Barabutis et al., 2020). GHRH agonists (e.g., JI-34, MR-409) promote angiogenesis by activating GHRHR on endothelial and progenitor cells, enhancing vascular endothelial growth factor (VEGF)-driven PI3K/Akt/mTOR signaling, and improving tissue perfusion in ischemic conditions (Florea et al., 2014; Xia et al., 2016). Conversely, antagonists (e.g., MIA-602) inhibit pathological angiogenesis in cancers by suppressing VEGF and VEGFR pathways (Munoz-Moreno et al., 2018). GHRH also regulates endothelial function, with IGF-1 mediating NO-dependent vasodilation (Wilson et al., 2021), while agonists like MR-409 directly improve vascular relaxation via antioxidant and anti-inflammatory effects (Ren et al., 2023). Furthermore, GHRH analogues protect against oxidative stress, inflammation, and apoptosis in cardiovascular diseases by modulating nuclear factor *κ*B (NF-κB), nuclear erythroid 2-related factor 2 (Nrf2), and PI3K/Akt pathways, reducing infarct size, and preventing vascular calcification (VC) (Granata et al., 2009; Dulce et al., 2025). Additionally, GHRH agonists enhance calcium handling in cardiomyocytes, improving contractility and relaxation in heart failure (Gesmundo et al., 2017) (Figure 2).

### 3.1 Regulating angiogenesis

Angiogenesis is the formation of new blood vessels from pre-existing vasculature. It begins with endothelial cell activation by pro-angiogenic factors such VEGF, fibroblast growth factor (FGF). GHRH promotes angiogenesis primarily through two mechanisms: (1) direct activation of GHRHR on vascular endothelial cells and stem cells (e.g., MSCs, EPCs) (Xia et al., 2016), and (2) indirect stimulation via the GH/IGF-1 axis (Drozd et al., 2025). It is well known that GHRH can stimulate pituitary GH release to induces hepatic IGF-1 production, which activates PI3K/Akt/mTOR signaling in endothelial and stromal cells, promoting VEGF transcription (Xia et al., 2016). It has been shown that IGF-1 knockdown reduces VEGF levels (Kondo et al., 2003), impairing angiogenesis in wound healing and cancer models. VEGF agonist enhances endothelial cell survival, migration, and vascular permeability. The PI3K/Akt pathway, triggered by IGF-1, is critical for endothelial cell proliferation and vessel formation, as evidenced by increased Akt phosphorylation following IGF-1 treatment (Zhang et al., 2024). Additionally, PI3K/Akt activation facilitates extracellular matrix (ECM) remodeling via matrix metalloproteinase (MMP)-2/9, releasing matrix-bound VEGF and reinforcing angiogenic responses. Thus, GHRH-driven IGF-1 signaling amplifies VEGF expression via PI3K/Akt, fostering angiogenesis (Lin et al., 2017).

GHRH or its agonists, such as JI-34, JI-38, also bind to GHRHR to trigger downstream signaling pathways, including STAT3, PI3K/Akt, and MAPK/ERK, which upregulate VEGF expression and secretion, and enhances endothelial cell survival, proliferation and migration (Ma et al., 2016). It has been shown that preconditioning MSCs with GHRH agonists significantly enhances their angiogenic potential, as evidenced by increased capillary density and blood flow recovery in ischemic limb models (Li et al., 2020). GHRH agonists exhibit potent pro-angiogenic effects, making them promising for treating ischemic conditions (Xia et al., 2016; Yu and Peng, 2025). Shao et al. (2026) reported that a modified long-acting hybrid GHRH analog enhanced the angiogenic potential of endothelial cells and, in the ischemic hindlimb model, improved blood perfusion and preserved limb function. In preclinical studies, GHRH-A JI-34-preconditioned MSCs showed elevated VEGF and stromal cell derived factor 1 (SDF-1) secretion, improved cell viability, and enhanced engraftment in ischemic tissues (Ma et al., 2016; Xia et al., 2016). These MSCs also activated STAT3 and Akt phosphorylation, leading to robust neovascularization and limb salvage in murine hindlimb ischemia models (Han et al., 2016). Similarly, GHRH-stimulated EPCs demonstrates increased migration and tube-forming capacity, accelerating perfusion recovery. The agonists’ ability to amplify paracrine signaling (e.g., VEGF, FGF) and recruit pericytes underscores their utility in regenerative medicine, particularly for myocardial infarction and critical limb ischemia (Granata et al., 2009; Li et al., 2020).

Conversely, GHRH antagonists (e.g., MIA-602, MIA604) inhibit tumor angiogenesis by blocking GHRHR signaling (Klukovits et al., 2012). They suppress VEGF release from cancer cells and endothelial cells, downregulate VEGFR/EGFR expression, and disrupt PI3K/Akt and FAK pathways, critical for EC survival and tumor vascularization. In cancers (e.g., ovarian, lung), these antagonists reduce microvessel density and impede metastatic spread (Klukovits et al., 2012; Zhang et al., 2020). For instance, MIA-602 decreased VEGF secretion in OVCAR-3 cells (Klukovits et al., 2012), while MZ-J-7-114 inhibited EGF/VEGFR-driven angiogenesis (Kanashiro et al., 2007). By targeting both the GH/IGF-1 axis and local GHRH autocrine loops, GHRH antagonists offer a dual anti-angiogenic strategy, highlighting their potential as adjuncts to conventional cancer therapies.

### 3.2 Regulating endothelial function

The vascular endothelium plays a crucial role in maintaining vascular homeostasis by regulating vasodilation, permeability, and inflammation. Endothelium-dependent relaxation is primarily mediated by nitric oxide (NO), which promotes smooth muscle relaxation and blood flow (Zhou et al., 2010). Additionally, the endothelial barrier selectively controls the passage of fluids and molecules, with its integrity maintained by tight junctions and adhesion proteins. Dysfunction in these processes, such as reduced NO bioavailability or increased permeability, contributes to vascular diseases, including atherosclerosis and diabetic microangiopathy (Pi et al., 2018).

The vasodilatory effects of GHRH remain poorly understood, which may involve both direct and indirect pathways, with IGF-1 serving as a key mediator of its vascular actions (Imrie et al., 2012; Wilson et al., 2021). Direct GHRH-mediated activation of endothelial nitric oxide synthase (eNOS) remains uncertain (Penna et al., 2013; Dulce et al., 2025), as GHRH-induced NO production primarily occurs in somatotrophs and cardiomyocytes via the sGC/cGMP/PKC pathway. In contrast, IGF-1, whose release is stimulated by GHRH through the GH axis, directly enhances eNOS activity, increasing NO bioavailability and promoting vascular smooth muscle relaxation (Wilson et al., 2021). IGF-1 also activates potassium channels (Biadgo et al., 2020), demonstrating robust NO-dependent and independent vasodilatory mechanisms. Notably, GHRH agonists like MR-409 improve endothelium-dependent relaxation and reduce vascular injury in diabetic models, independent of GH/IGF-1, likely through antioxidant and anti-inflammatory mechanisms (Ren et al., 2023).

GHRH exhibits context-dependent vascular effects that extend beyond IGF-1 mediation. While IGF-1 consistently promotes vasorelaxation through multiple pathways, GHRH itself influences vascular structure by enhancing angiogenesis via endothelial progenitor cell activation, as seen in heart failure models. GHRH agonists like MR-409 improve vascular function through antioxidant and anti-inflammatory effects, independent of GH/IGF-1 in some cases (Dulce et al., 2025). Conversely, GHRH antagonists protect against endothelial barrier disruption by reducing oxidative stress and inflammation (Fakir and Barabutis, 2024), suggesting a dual role for GHRH signaling in vascular homeostasis. Together, these findings position GHRH as a modulator of vascular tone with both direct (e.g., angiogenic) and indirect (IGF-1-mediated NO production) effects, whereas IGF-1 serves as the primary executor of NO-driven vasodilation and endothelial protection (Biadgo et al., 2020).

Both GHRH and IGF-1 play critical roles in regulating endothelial barrier integrity and permeability (Drozd et al., 2025; Dulce et al., 2025). GHRH agonists, such as JI-34 and MR-409, stabilize the endothelial barrier by reducing myosin light chain phosphorylation and enhancing cAMP-dependent signaling, thereby counteracting hyperpermeability (Lucas et al., 2012). MR-409 further protects against vascular leakage by upregulating Nrf2 to suppress VEGF and inflammation, particularly in diabetic retinopathy (Thounaojam et al., 2017). Conversely, GHRH antagonists preserve barrier function by inhibiting STAT3/ERK activation and ROS generation, as demonstrated in models of pulmonary endothelial injury (Xia et al., 2016). These findings suggest that GHRH agonists or antagonists can therapeutically target endothelial dysfunction in metabolic and inflammatory disorders (Drozd et al., 2025).

Similarly, IGF-1 maintains endothelial barrier stability by preserving intercellular junctions and reducing oxidative stress (Drozd et al., 2025). Deficiency in IGF-1 signaling disrupts tight junction proteins, increasing permeability and accelerating atherosclerosis (Higashi et al., 2020). Clinically, low IGF-1 levels correlate with higher cardiovascular risk, highlighting its protective role in vascular health (Wang et al., 2023). Together, GHRH and IGF-1 signaling pathways offer promising therapeutic strategies for enhancing endothelial barrier function and mitigating vascular disease progression.

### 3.3 Anti-inflammatory, anti-oxidant and anti-apoptotic effects

Cardiovascular diseases are driven by oxidative stress, inflammation, and apoptosis, which collectively contribute to endothelial dysfunction, atherosclerosis, and myocardial injury (Kang et al., 2024). ROS disrupt cellular homeostasis by oxidizing lipids, proteins, and DNA, impairing NO bioavailability and promoting vascular stiffness. Chronic inflammation, mediated by cytokines tumor necrosis factor-α (TNF-α), interleukin-6 (IL-6), macrophage activation, and NF-κB signaling, exacerbates plaque instability and thrombosis (Kang et al., 2024; Abrashev et al., 2025). These processes further induce apoptosis in endothelial cells, cardiomyocytes, and vascular smooth muscle cells (VSMCs), accelerating cardiac remodeling and heart failure. Mitochondrial dysfunction, caspase activation, and dysregulation of Bcl-2 family proteins amplify cell death, worsening ischemic injury.

GHRH analogues, including the antagonist MIA-690 and agonist MR-409, exhibit potent anti-inflammatory, antioxidant, and anti-apoptotic effects, offering therapeutic potential in CVDs (Recinella et al., 2020; Granata et al., 2025). In LPS-induced inflammation models, these peptides suppress NF-κB, TNF-α, and IL-6 while upregulating Nrf2, a key regulator of antioxidant defenses (Recinella et al., 2020). GHRH antagonists (e.g., MIA-602) further mitigate oxidative stress by reducing ROS, inducible nitric oxide synthase, and cyclooxygenase-2 in conditions like uveitis, lung injury, and prostate hyperplasia. These effects are mediated through inhibition of NF-κB, STAT3, and MAPK pathways, highlighting the role of GHRH in modulating inflammatory cascades (Liang et al., 2020).

Beyond inflammation, GHRH exerts direct anti-apoptotic effects in cardiac myocytes exposed to ischemic or adrenergic stress. By activating GHRHR signaling, GHRH triggers PI3K/Akt and ERK1/2 pathways, suppressing pro-apoptotic factors like inducible cAMP early repressor (ICER) and enhancing cell survival. In ischemia-reperfusion (I/R) injury models, GHRH improves cardiac recovery, reduces infarct size, and attenuates fibrosis, independent of GH/IGF-1 axis activation (Granata et al., 2009). Similar mechanisms extend to VC, where MR409 inhibits ROS/NADPH oxidase activity and downregulates osteogenic markers (Runx2, ALP) in VSMCs via cAMP/PKA and PI3K/Akt pathways, thereby preventing VC progression (Shen et al., 2018).

GHRH’s anti-inflammatory and antioxidant properties also apply to neurovascular disorders. In ischemic stroke (IS), MR409 resolves chronic inflammation by modulating Jak-STAT and Toll-like receptor pathways, promoting neurogenesis and tissue repair (Liu et al., 2021). Additionally, in severe COVID-19, the antagonist MIA-602 blunts cytokine storms (TNF-α, IL-1β) and oxidative stress in immune cells, highlighting its broad-spectrum anti-inflammatory potential (Granato et al., 2023). Collectively, GHRH and its analogues modulate oxidative stress, inflammation, and apoptosis through multi-targeted mechanisms, making them promising candidates for treating atherosclerosis, heart failure, VC, and stroke.

### 3.4 Regulating calcium handling in the process of cardiac excitation-contraction coupling

GHRH analogs exert significant effects on calcium handling in cardiomyocytes, improving excitation-contraction coupling in heart failure models (Dulce et al., 2023). Studies in pressure-overloaded mice treated with the GHRH agonist MR-409 demonstrate enhanced cardiomyocyte contractility, characterized by faster calcium transient rise and improved sarcolemmal structure, including T-tubule organization (Dulce et al., 2025). While MR-409 restores sarcoplasmic/endoplasmic reticulum calcium ATPase 2α (SERCA2α) expression, a key regulator of calcium reuptake into the sarcoplasmic reticulum (SR), it paradoxically slows calcium decay, likely due to reduced phosphorylation of phospholamban (PLB) at threonine 17, which inhibits SERCA2α activity (Dulce et al., 2023). Conversely, in Ang II-induced heart failure with preserved ejection fraction (HFpEF) models, the GHRH agonist MR-356 accelerates calcium decay and sarcomere relaxation by increasing PLB phosphorylation (at serine 16 and threonine 17) and reducing abnormal SR calcium leak through ryanodine receptor 2 (RyR2) stabilization (Dulce et al., 2023). These findings suggest that GHRH signaling fine-tunes calcium cycling by modulating SERCA2α-PLB interaction and RyR2 function, thereby enhancing both systolic and diastolic performance.

Additionally, GHRH agonists improve myofilament calcium sensitivity by regulating phosphorylation of key contractile proteins, including cardiac troponin I (cTnI), myosin-binding protein C (MyBPC), and titin. In HFpEF models, MR-356 restores phosphorylation of these proteins, promoting efficient cross-bridge cycling and reducing diastolic stiffness (Dulce et al., 2023). The combined effects on calcium handling and sarcomere function highlight GHRH’s role in preserving cardiomyocyte contractility and relaxation, making it a promising therapeutic target for heart failure with both reduced and preserved ejection fraction.

## 4 Therapeutic potential of GHRH and GHRH analogues in cardiovascular diseases

Recent studies highlight the potential of GHRH analogues in mitigating key pathological processes underlying CVDs, including VC, ischemic heart disease (IHD), HF, and age-related cardiovascular decline (Bagno et al., 2015; Xiang et al., 2021; Ren et al., 2023). Unlike traditional GH or IGF-1 therapies, GHRH analogues exert pleiotropic effects: anti-inflammatory, antioxidant, and antifibrotic, while bypassing the adverse consequences of systemic GH/IGF-1 elevation. Preclinical studies demonstrate their ability to inhibit osteogenic transformation in VSMCs, reduce infarct size post-myocardial infarction, improve cardiac remodeling, and counteract age-related mitochondrial dysfunction (Shen et al., 2018; Schally et al., 2019). These benefits are mediated via direct GHRHR activation, suggesting a targeted approach with a favorable safety profile.

### 4.1 Vascular calcification

VC is an active, pathological process driven by the osteogenic transformation of VSMCs, promoted by hyperphosphatemia, chronic inflammation, oxidative stress, and deficiencies in calcification inhibitors (Vieceli Dalla Sega et al., 2022). These factors lead to calcium-phosphate deposition in arterial walls, mimicking bone formation. The resulting stiffening of vessels increases cardiovascular risk, contributing to hypertension, reduced coronary perfusion, and atherosclerotic plaque instability (Lanzer et al., 2021). VC is particularly prevalent in aging, diabetes, and chronic kidney disease, where it independently predicts adverse outcomes, including myocardial infarction, stroke, and cardiovascular mortality (Lanzer et al., 2021).

Despite its clinical importance, treatment options remain limited. Phosphate binders and vitamin K supplementation show modest benefits but fail to reverse established calcification (Saunders et al., 2024). Statins and bisphosphonates have inconsistent effects (Shahraki et al., 2023), while novel therapies targeting osteogenic signaling (e.g., Runx2 inhibition) are still under experimental stage. The complexity of calcification pathways, coupled with the need to balance mineral metabolism without disrupting bone health, poses significant therapeutic hurdles (Lanzer et al., 2021). Improved biomarkers and targeted therapies are urgently needed to address this growing cardiovascular burden (Vieceli Dalla Sega et al., 2022).

Increasing evidence has suggested that endogenous GHRH play a role in regulating bone and VC through stimulating GH/IGF-1 production (Di Bartolo et al., 2011). IGF-1 within physiological concentration exerts anti-inflammatory and anti-oxidative effects, potentially inhibiting VSMC transdifferentiation into osteoblast-like cells, IGF-1 deficiency can impair vascular repair, exacerbating the progress of VC (Di Bartolo et al., 2011). However, dysregulation of GHRH/GH/IGF-1 axis, particularly GH excess, may accelerate VC by increasing calcium-phosphate deposition and osteogenic markers in VSMCs.

It has been shown that GHRH analogues exhibit pharmacological properties including anti-inflammatory, antioxidant, and lipid-modulating effects (Recinella et al., 2020), as well as suppression of osteogenic gene expression. Shen et al. (2018) demonstrated that in an osteoprotegerin-deficient mouse model, subcutaneous administration of MR-409 for 4 weeks significantly inhibited aortic calcification by reducing alkaline phosphatase (ALP) activity and suppressing the expression of osteogenic markers, such as runt-related transcription factor 2 (Runx2), osteonectin, and osteocalcin. In osteogenic medium-induced human and mouse aortic SMC models, MR-409 inhibited NADPH oxidase activity, reduced ROS production, and promoted NF-κB (p65) dephosphorylation via activation of the cAMP/PKA pathway, thereby suppressing the NF-κB-Runx2/ALP-mediated osteogenic process and attenuating VC (Panizo et al., 2009; Shen et al., 2018). Recently, we (Ren et al., 2023) have demonstrated that MR-409 treatment for 8 weeks effectively inhibits VC and cardiac valve calcification, associated with improving endothelium-dependent vasorelaxation and reducing vascular injury in db/db diabetic mice. Notably, in all studies above mentioned, MR-409 did not alter serum GH levels in either of these VC animal models, suggesting that anti-calcification effects of MR409 may be mediated via activating GHRH receptor signaling in vascular cells (Ren et al., 2023), and GHRH analogues especially for MR409, represent a promising but underexplored therapeutic avenue for VC.

### 4.2 Ischemic heart disease (IHD)

IHD is caused by reduced blood flow to the heart muscle, primarily due to atherosclerosis of the coronary arteries. This leads to myocardial ischemia, manifesting as angina pectoris or, in severe cases, myocardial infarction (MI) (Butler et al., 2024). Chronic IHD can result in heart failure, arrhythmias, and sudden cardiac death. Risk factors include hypertension, diabetes, smoking, and hyperlipidemia. IHD remains a leading global cause of morbidity and mortality (Hausenloy and Yellon, 2013). Treatment involves lifestyle modifications, medications (antiplatelets, statins, beta-blockers), and revascularization (stents or bypass surgery). Emerging therapies target inflammation, metabolic pathways, and regenerative repair to improve outcomes (Mastoor et al., 2025).

The role of endogenous GHRH/GH/IGF-1 axis in ischemic myocardial disease remains controversial, with studies reporting both beneficial and detrimental effects. Bollano et al. (2000) demonstrated that chronic GH overexpression in transgenic mice led to mitochondrial dysfunction and impaired cardiac performance, suggesting long-term GH excess may harm the heart. Conversely, other studies indicate GH therapy can attenuate left ventricular remodeling in post-MI, reducing dilation, improving systolic/diastolic function, and enhancing contractility independently of IGF-1 (Daskalopoulos et al., 2015). However, GH treatment also risks adverse effects like cardiac hypertrophy and metabolic disturbances (Sesti et al., 2007). Notably, GHRH agonists (e.g., JI-38) show cardioprotective benefits, preserving LV function, reducing apoptosis, and stimulating cardiac precursor cell proliferation without elevating systemic GH/IGF-1 levels (Bagno et al., 2015). This suggests direct GHRH receptor activation in cardiomyocytes may promote repair, bypassing GH-related side effects. While GH’s efficacy depends on context (e.g., dosing, timing).

**FIGURE 3:**
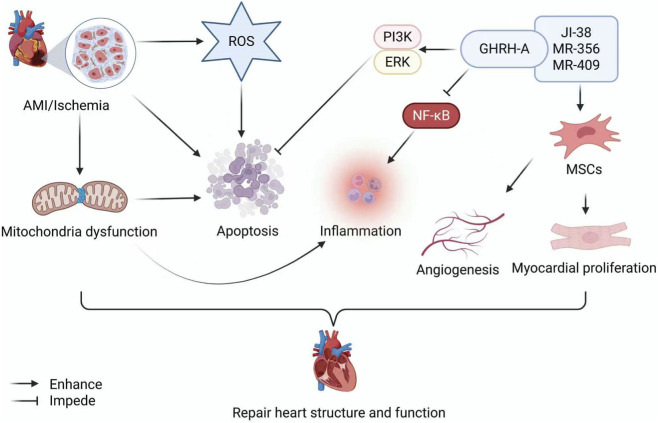
The proposed mechanisms by which GHRH agonists (JI-38, MR-356 & MR-409) in ameliorating acute myocardial infarction (AMI)-induced cardiac dysfunction and injury. GHRH agonists bind to GHRH receptors in cardiomyocyte to activate PI3K and ERK1/2 pathway. This activation inhibits cardiomyocyte apoptosis and nuclear factor *κ*B (NF*κ*B) inflammatory pathway, reducing reactive oxygen species (ROS) generation, and improving AMI-induced mitochondrial dysfunction. Furthermore, GHRH agonists can also promote cardiac repair through multiple mechanisms involving mesenchymal stem cells (MSCs). These include:1) enhanced MSC-mediated angiogenesis via upregulation of vascular endothelial growth factor (VEGF); 2) stimulating MSC differentiation into functional cardiomyocytes; and 3) promotion of endogenous cardiomyocyte proliferation. Together, these mechanisms synergistically contribute to structural and functional cardiac recovery post-injury.

Preclinical studies demonstrate that synthetic GHRH-A exert significant cardioprotective effects in animal models of acute myocardial infarction (AMI) (Kanashiro-Takeuchi et al., 2012; Bagno et al., 2015). In rat models with permanent coronary ligation, treatment with GHRH-A (JI-38, MR-356, MR-409) for 4 weeks reduced infarct size, improved cardiac function, and attenuated adverse remodeling by enhancing cardiac stem cell proliferation, angiogenesis, and antiapoptotic signaling (Granata et al., 2009; Kanashiro-Takeuchi et al., 2012). These effects were mediated through GHRHR activation, as evidenced by the reversal of benefits with GHRH antagonists. Notably, porcine MI models treated with MR-409 showed reduced scar size and improved diastolic function, accompanied by increased GHRHR expression in the infarct border zone (Bagno et al., 2015). Importantly, GHRH-A’s cardioprotective effects were independent of systemic GH/IGF-1 elevation (Cai et al., 2014; Daskalopoulos et al., 2015), avoiding the adverse effects associated with GH therapy, such as fluid retention and metabolic disturbances (Figure 3).

GHRH-A promotes myocardial repair through multiple pathways. *In vitro* studies reveal that GHRH-A activates prosurvival signaling cascades, including PI3K/Akt, ERK1/2, and cAMP/PKA/CREB, reducing cardiomyocyte apoptosis and improving cell survival (Granata et al., 2009). *In vivo*, GHRH-A enhances cardiac progenitor cell recruitment, stimulates cell proliferation, and increases capillary density, contributing to functional recovery (Bagno et al., 2015; Gesmundo et al., 2017). Additionally, GHRH-A modulates inflammatory and fibrotic responses by reducing pro-inflammatory cytokines, such as IL-6, TNF-α, and inhibiting pro-fibrotic pathways (Gallo et al., 2015), while upregulating bone morphogenetic proteins. These actions collectively mitigate adverse remodeling and improve hemodynamic parameters, such as ejection fraction and ventricular pressure. The upregulation of GATA binding protein-4, a key transcription factor of hypertrophy-associated genes, further underscores the role of GHRH-A in adaptive cardiac responses.

GHRH-A outperforms GH or IGF-1 therapy in preclinical studies, demonstrating consistent benefits across rodent and swine MI models (Granata et al., 2009; Kanashiro-Takeuchi et al., 2012; Bagno et al., 2015). While rodent studies highlight improvements in systolic function and infarct size reduction, swine models emphasize diastolic function enhancement, reflecting species-specific remodeling differences (Bagno et al., 2015). The absence of GH/IGF-1-related side effects positions GHRH-A as a safer alternative for IHD treatment. Its ability to target multiple repair mechanisms, including anti-inflammatory, antifibrotic, and regenerative effects, makes it a potential therapeutic candidate for AMI. The current evidence supports GHRH-A’s potential as a novel treatment for AMI and ischemic cardiomyopathy (Schally et al., 2019; Dulce et al., 2025).

### 4.3 Heart failure

HF is a progressive clinical syndrome characterized by impaired cardiac function. HF can be classified by left ventricular ejection fraction (LVEF) into two main types: HF with reduced ejection fraction (HFrEF, LVEF ≤40%), characterized by impaired systolic function and weakened myocardial contraction; and HF with preserved ejection fraction (HFpEF, LVEF ≥50%), marked by diastolic dysfunction and reduced ventricular compliance (Hahn et al., 2024; Lilly et al., 2025).

It is well known that cardiac structure and performance are significantly affected by endocrine system. The GHRH/GH/IGF-1 axis can regulate cardiac performance through dual endocrine/paracrine mechanisms, acting via cardiomyocyte receptors to enhance contractility through calcium handling (SERCA2α activation via PI3K/Akt) and adaptive hypertrophy (JAK2/STAT5 pathways) (Dulce et al., 2023). In HF, this axis demonstrates paradoxical effects: while animal models and GH-deficient patients show improved systolic function with therapy, clinical trials reveal limited efficacy due to acquired GH resistance in advanced HF (Lombardi et al., 1997; Marra et al., 2025). The system’s compensatory upregulation during hemodynamic stress is counterbalanced by risks of maladaptive remodeling, chronic excess promotes fibrosis and diastolic dysfunction, particularly in HFpEF. Although GH/IGF-1 improves metabolic parameters and vascular resistance, clinical application is hampered by their side effects, including arrhythmogenic potential, fluid retention, and heterogeneous patient responses (Carroll and Van den Berghe, 2001). Current evidence suggests that endogenous GHRH/GH/IGF-1 has limited therapeutic efficacy in HF with a narrow therapeutic window (Lombardi et al., 1997). Targeted therapy for GH-deficient HFrEF patients or administration of downstream IGF-1 agonists may potentially provide certain therapeutic benefits while minimizing systemic side effects.

GHRH analogues demonstrate multi-targeted cardioprotective effects by modulating calcium handling, inflammation, and fibrosis (Kanashiro-Takeuchi et al., 2015; Gesmundo et al., 2017; Dulce et al., 2023). Using a murine model of transverse aortic constriction (TAC)-induced pressure overload, which is a HFrEF model, Gesmundo et al. (2017) demonstrated that MR-409 attenuated cardiac hypertrophy and improved cardiac function by blocking Gq signaling and activating the cAMP/PKA pathway, thereby counteracting myocardial infarction-induced cardiac hypertrophy, fibrosis and remodeling. For HFpEF in either murine models with Ang-II infusion or high-fat diet the NOS inhibitor N(ω)-nitro-L-arginine methyl ester treatment, MR-356 reduced cardiomyocyte hypertrophy and fibrosis via GHRHR-dependent suppression of transforming growth factor (TGF)-β, monocyte attractant protein (MCP)-1, and α-smooth muscle actin (α-SMA), while enhancing diastolic function and calcium kinetics (Dulce et al., 2023). These effects are largely GH/IGF-1-independent, as evidenced by direct cAMP/cGMP elevation in cardiomyocytes.

In HFpEF, GHRH-A show divergent structural effects: MR-356 abolished cellular hypertrophy *ex vivo* but did not reverse ventricular mass *in vivo*. Nonetheless, all models consistently demonstrated anti-fibrotic and anti-inflammatory actions, with MR-409 additionally improving mitochondrial function in aging-related HF and a porcine cardiorenal model (Bagno et al., 2015; Xiang et al., 2021). Notably, blood pressure modulation remains inconsistent, MR-356 prevented Ang-II-induced hypertension but failed to lower pressure in established cardiometabolic HFpEF (Dulce et al., 2023).

GHRH-A address unmet needs in both systolic HFrEF and diastolic HFpEF, particularly in comorbidities like chronic kidney disease (Rieger et al., 2021). Their ability to improve pro-BNP levels, calcium handling, and exercise capacity highlights translational potential. However, optimal dosing and patient stratification require further study, especially in elderly populations where inflammatory modulation may be pivotal. Large-animal data underscore relevance to human pathophysiology, warranting clinical trials to validate efficacy and safety.

### 4.4 Age-related cardiovascular decline

Aging is a major risk factor for CVDs, with the decline in GHRH/GH/IGF-1 axis (somatopause) contributing to cardiovascular dysfunction through impaired mitochondrial function, increased oxidative stress, and fibrosis, leading to diastolic dysfunction and vascular stiffness (Banks et al., 2010; Tausendfreund et al., 2024). The loss of cardioprotective IGF-1 and GH effects exacerbates inflammation, endothelial dysfunction, and metabolic dysregulation, worsening cardiac remodeling and atherosclerosis progression, making this axis as a key therapeutic target for age-related CVDs (Biadgo et al., 2020; Higashi et al., 2020). However, direct GH/IGF-1 replacement carries risks like fluid retention, GHRH analogues offer a promising alternative by activating GHRH receptor signaling without significantly elevating systemic GH/IGF-1 (Xiang et al., 2021; Dulce et al., 2023).

GHRH analogues demonstrate significant promise in addressing age-related cardiovascular decline through multiple protective mechanisms (Banks et al., 2010; Xiang et al., 2021). In preclinical studies, compounds like JI-34 (10–100 nmol/L) and MR-356 (500 nmol/L) showed remarkable cardioprotection against oxidative stress, reducing ROS production in irradiated cardiomyocytes by activating RISK/SAFE survival pathways (Kiscsatari et al., 2016). More impressively, MR-409 (10 µg/day, sc) administered over 6 months to aged mice produced comprehensive improvements, including enhanced systolic and diastolic function, increased exercise capacity, and even visible anti-aging effects like hair regrowth (Xiang et al., 2021). These benefits are mediated through GHRHR/cAMP/PKA signaling, which restores mitochondrial health by increasing the long-to-short OPA1 isoform ratio, improving oxidative phosphorylation, and reducing aberrant mitophagy. Complementary *in vitro* studies using doxorubicin-stressed H9c2 cardiomyocytes further confirmed these mitochondrial protective effects (Xiang et al., 2021), suggesting GHRH analogues could prevent both acute cardiac injury and chronic age-related deterioration.

Beyond cardiomyocyte protection, GHRH analogues address key pathological features of cardiovascular aging. They counteract mitochondrial dysfunction by upregulating OPA1 isoforms and reducing ROS accumulation in aged hearts. Simultaneously, they attenuate myocardial fibrosis by suppressing TGF-β and α-SMA signaling, thereby improving diastolic compliance in conditions like HFpEF (Dulce et al., 2025). In vascular tissues, these analogues inhibit osteogenic transdifferentiation of smooth muscle cells by downregulating Runx2 and alkaline phosphatase, potentially preventing age-related arterial stiffening and calcification (Shen et al., 2018). Importantly, MR-409 achieves these effects without elevating systemic GH/IGF-1 level, avoiding the fluid retention and metabolic disturbances associated with direct GH therapy. This GH/IGF-1-independent action makes GHRH analogues particularly suitable for elderly patients with multiple comorbidities.

GHRH analogues offer a multifaceted approach to preventing and treating age-related cardiovascular decline. MR-409 demonstrated benefits in improving cardiac function, exercise tolerance (Rieger et al., 2021), and even systemic aging markers position it as a leading candidate for human trials. Given their ability to simultaneously target mitochondrial dysfunction, fibrosis, inflammation, and VC, GHRH analogues represent a novel and comprehensive therapeutic strategy against cardiovascular aging. Clinical trials are warranted to validate these promising preclinical findings and establish their efficacy in human age-related cardiovascular diseases.

## 5 GHRH and its analogues in cerebrovascular diseases

### 5.1 GHRH receptor expression in the CNS and its neural protective effects

GHRH and GHRHR are expressed in the central nervous system (CNS), extending beyond their classical role in the hypothalamic-pituitary axis (Liu et al., 2025). In addition to the pituitary, GHRHR mRNA and protein are detected in the cerebral endothelial cells, neural stem cells, cortex, hippocampus, and brain tumors (Liao et al., 2010; Mezey et al., 2014; Liu et al., 2021), where they function in a paracrine manner to regulate neural activity (Mezey et al., 2014). The GHRHR activates cAMP/PKA/CREB signaling in pituitary somatotrophs to stimulate GH release, but in extrapituitary brain regions, it also engages PI3K/Akt and ERK pathways, influencing neuroprotection and plasticity (Halmos et al., 2025). Notably, synthetic GHRH analogues (GHRH-As), such as the JI and MR series (e.g., JI-34, MR-409), exhibit enhanced stability and receptor affinity, enabling targeted modulation of CNS function (Liu et al., 2025). These analogues can either stimulate or block GHRHR in neural tissues, offering therapeutic potential for CNS disorders by restoring imbalances in the GHRH/GH/IGF-1 axis (Liu et al., 2025). GHRH exerts neuroprotective effects through multiple mechanisms, including anti-apoptotic, antioxidant, and anti-inflammatory actions. In models of ischemic stroke (IS), and diabetic retinopathy (Jaszberenyi et al., 2012; Thounaojam et al., 2017; Liu et al., 2021; Pedrolli et al., 2026), GHRH agonists mitigate neuronal damage by suppressing NF-κB, TNF-α, and ROS, while upregulating Nrf2-mediated antioxidant defenses. They also promote neurogenesis in the hippocampus via CREB/brain derived neurotrophic factor (BDNF) signaling and stabilize synaptic function. For instance, MR-409 has shown efficacy in reducing ischemic brain injury and improving cognitive deficits, independent of systemic GH/IGF-1 effects (Liu et al., 2021). Conversely, GHRH antagonists may paradoxically protect neural tissues by inhibiting pathological angiogenesis or inflammation in conditions like glioblastoma (Kanashiro et al., 2005). The presence of GHRHR in ocular tissues further highlights its role in diabetic retinopathy, where agonists preserve retinal integrity by counteracting oxidative stress. In models of Alzheimer’s disease, MR-409 reduced Aβ deposition, tau phosphorylation, neuroinflammation, and neuronal/synaptic loss while improving cognitive performance by activating cAMP/PKA/CREB, ERK1/2, and PI3K/Akt pathways and upregulating NRF2 expression without altering systemic GH and IGF1 levels (Pedrolli et al., 2026). Together, these findings position GHRHR modulation as a promising strategy for neurodegenerative, cerebrovascular, and retinal diseases.

### 5.2 GHRH and its analogues in IS

IS remains a leading cause of mortality and permanent disability, characterized by acute neuronal necrosis due to hypoxia, inflammation, and oxidative stress. Current single-drug therapies often fail to address the complex ischemic cascade, which involves excitotoxicity, calcium overload, and apoptosis, leading to irreversible neurological deficits (Qin et al., 2022). Notably, IS patients frequently exhibit dysfunction of the GH/IGF-1 axis, with low plasma IGF-1 levels correlating with poor prognosis. Preclinical studies suggest IGF-1 supplementation can mitigate neuronal loss and improve functional recovery, highlighting the therapeutic potential of targeting this axis (Denti et al., 2004). However, the endogenous repair process, activation of neural stem cells in the subventricular zone and hippocampal subgranular zone is often inadequate in elderly patients due to a hostile ischemic microenvironment and diminished regenerative capacity (Qin et al., 2022).

IS presents a critical therapeutic challenge due to its complex pathophysiology involving neuronal necrosis, oxidative stress, and neuroinflammation (Hilkens et al., 2024). Current treatments fail to address the dual needs of neuroprotection and neuroregeneration (Hilkens et al., 2024). Recent preclinical studies demonstrate that GHRH analogues, particularly MR-409, offer promising multimodal benefits in IS models. Recently we (Liu et al., 2021) have shown that in transient middle cerebral artery occlusion (tMCAO) mice, subcutaneous MR-409 (5–10 μg/day) significantly reduced mortality, attenuated hippocampal atrophy, and improved functional recovery. These effects were mediated through GHRHR activation in NSCs, enhancing proliferation via Akt/CREB and BDNF/tropomyosin receptor kinase B (TrkB) pathways while suppressing apoptosis. Importantly, MR-409 improved the ischemic microenvironment by reducing inflammation and oxidative stress, enabling better NSC survival and differentiation into functional neurons in subventricular and subgranular zones.

A key consideration for GHRH-As therapy is blood-brain barrier (BBB) penetration. While native GHRH has limited CNS access, modified analogues show improved permeability. Jaeger et al. (2005) using iodinated GHRH antagonist JV-1–42 revealed a brain uptake rate of 0.85 μL/g/min after intravenous administration, with 0.41% of the dose accumulating in brain tissue. Although transport occurs through non-saturable mechanisms, efflux involves P-glycoprotein, suggesting partial BBB penetration. Importantly, subcutaneous MR-409s efficacy in tMCAO models implies sufficient CNS bioavailability, possibly through: (1) enhanced stability against peptidases, (2) partial BBB penetration during ischemic opening, and (3) action on circumventricular organs with subsequent secondary signaling. These demonstrated neuroprotective effects, including reduced infarct volume and improved NSC proliferation, confirm biologically active concentrations reach critical brain regions.

These findings position GHRH-As as unique candidates bridging acute neuroprotection and chronic neuroregeneration in IS. While MR-409 shows particular promise in modulating NSC dynamics, further pharmacokinetic studies should quantify brain concentrations across different administration routes and optimize delivery strategies (e.g., nanocarriers) to enhance CNS penetration. The combined evidence of efficacy in tMCAO models and measurable BBB transit supports clinical translation potential for GHRH-As in stroke recovery.

## 6 Clinical translational perspectives and conclusion

Extensive preclinical studies demonstrate significant therapeutic potential of GHRH analogues, particularly the agonist MR-409, for cardiovascular and cerebrovascular diseases (Dulce et al., 2025; Liu et al., 2025). Their benefits are attributed primarily to the direct activation of local GHRHRs, as evidenced by a lack of significant elevation in plasma GH/IGF-1 levels in several key studies (Bagno et al., 2015; Granata et al., 2025). To definitively separate these effects from the systemic GH/IGF-1 axis, future research should confirm in target tissues: 1) whether local activation of GHRH-dependent PKA/Akt/CREB pathway post-administration is independent of IGF-1R signaling, and 2) how responses mediated by GHRH receptor splice variants (e.g., SV1) across different tissues. These studies will provide molecular base to support the hypothesis.

**FIGURE 4:**
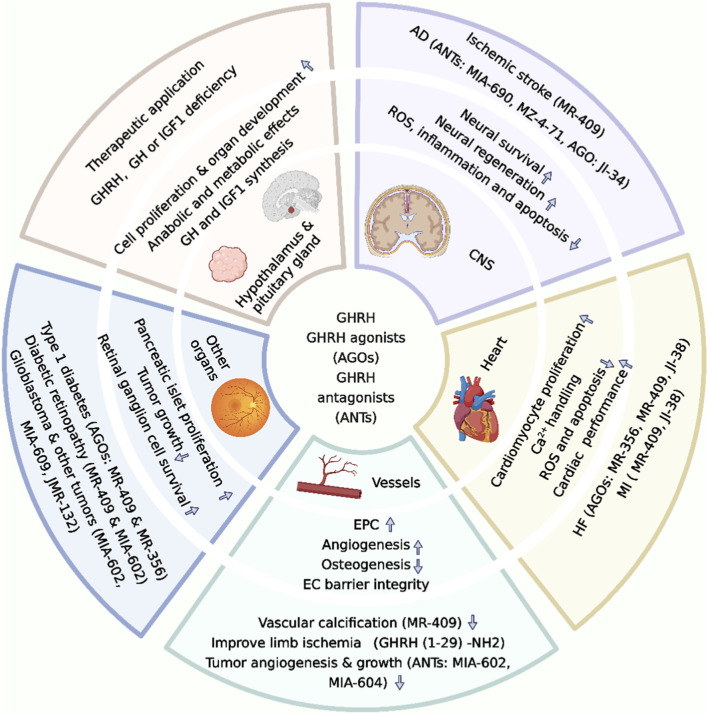
Growth hormone-releasing hormone (GHRH) and its analogues in the treatment of cardiovascular and cerebrovascular diseases. AGOs: agonists; ANTs: antagonists; CNS: central nervous system.

In the cardiovascular realm, MR-409 mitigates VC by suppressing osteogenic markers via the cAMP/PKA signaling pathway while improving endothelial function (Shen et al., 2018). Concurrently, it enhances cardiac repair post-MI by promoting VEGF/PI3K/Akt-mediated angiogenesis, reducing infarct size and improving left ventricular function in rodent and porcine models (Bagno et al., 2015). Furthermore, MR-356 improves diastolic function in HFpEF models by normalizing calcium handling and attenuating fibrosis, whereas MR-409 reverses pathological hypertrophy in HFrEF (Dulce et al., 2023). Chronic MR-409 treatment in aged mice also restores mitochondrial function and reduces cardiac stiffness (Banks et al., 2010). In IS models, MR-409 demonstrates neuroprotective and regenerative effects, reducing mortality by 50%, preserving hippocampal volume, and stimulating neural stem cell proliferation via BDNF/TrkB-CREB pathways (Liu et al., 2021). These analogues also show neuroprotection in retinal ganglion cell injury models. This pleiotropic efficacy, encompassing anti-calcification, pro-angiogenic, anti-fibrotic, and neuroregenerative mechanisms, positions GHRH analogues as promising candidate therapeutics for cardiovascular and cerebrovascular diseases with significant unmet needs (Dulce et al., 2025; Liu et al., 2025) (Table 1; Figure 4).

**TABLE 1:** Therapeutic cardiovascular and cerebrovascular beneficial effects of GHRH analogues in major preclinical studies.

Diseases model or cell type	GHRH type and code	Potential therapeutic effects and pharmacological targets	Refs
VC			
Osteogenically Differentiated aortic SMCs	Agonist MR-409 Antagonist MIA-602	Inhibition of ROS, RANKL-RUNX2 pathway Inhibition of VSMC calcification Promoting VSMC calcification	Shen et al. (2018)
OPG^−/−^ mice	Agonist MR-409	Antioxidant, Anti-inflammation, reduced VC Inhibition of NADPH oxidase, NF-κB and RUNX2	Shen et al. (2018)
Db/Db mice	Agonist MR-409	Reducing VC and improving EDR Inhibition of ROS, RUNX2, upregulation of Klotho	Ren et al. (2023)
MI			
I/R myocardial injury and ISO-induced apoptosis	GHRH and Antagonist JV-1–36	Reducing I/R injury, ISO-induced apoptosis via activating ERK1/2 &PI3K pathway, reversed by ANT-1–36	Granata et al. (2009)
Permanent LAD Ligation in rats	Antagonist JI-38	Enhance CSC, EPC proliferation, reduces MI size and CM apoptosis, and improve heart function and hemodynamics	Kanashiro-Takeuchi et al. (2012)
Porcine transient LAD occlusion model	Agonist MR-409	Reducing MI scar mass and size, improving LV diastolic strain and hemodynamics in large animal swine	Bagno et al. (2015)
HF			
Ang II model with HFpEF in mice	Agonist MR-356	Reversed Ang II-induced LV hypertrophy, fibrosis diastolic dysfunction via GHRHR-cAMP pathway	Dulce et al. (2023)
TAC-induced HF	Agonist MR-409	Mitigation of TAC-induced HF, improvement of ventricular contractility and sarcolemmal structure	Gesmundo et al. (2017)
Aged mice with Cardiometabolic HF	Agonist MR-409 and MR-356	Improvement in metabolic and heart function, exercise capacity, reduced cardiac inflammation	Kanashiro-Takeuchi et al. (2023)
CKD-induced HF (5/6 nephroctomy)	Agonist MR-409	Improvement in LV structure, hemodynamics, diastolic function and molecular markers	Rieger et al. (2021)
AMI-induced HF	Agonist MR-409, JI-38, MR-356	Reduce MI size, inhibit inflammation and improve cardiac function and remodeling	Kanashiro-Takeuchi et al. (2015)
IS			
OGD-induced NSC injury	Agonist MR-409	Enhance proliferation and inhibit apoptosis	Liu et al. (2021)
tMCAO-operated mice	Agonist MR-409	Reduce mortality, ischemic insult, improve neurological functionary recovery, enhance NSC-derived neural regeneration via Akt/CREB and BDNF/TrkB pathways	Liu et al. (2021)
Others			
Radiation-exposed NRVMs	Agonist MR-409	Improvement in exercise, cardiac function, mitochondria function, survival rate	Kiscsatari et al. (2016)
Doxorubicin-induced CM injury	Agonist MR-409	Reduced ROS, cell senescence	Xiang et al. (2021)
Senescent mouse myocardium Doxorubicin-induced H9C2 CMs	Agonist MR-409	Improvement systolic cardiac function, mitochondrial function via GHRHR-PKA	Xiang et al. (2021)
GHRH-pretreated MSCs	Agonist JI-38, MR-409 and MR-356	Enhance CSC proliferation, survival, renewal and myocardial repair	Florea et al. (2014)
PE-induced CM hypertrophy	Agonist MR-409	Decrease hypertrophy gene expression via Gαs/cAMP/PKA pathway	Thounaojam et al. (2017)

GHRH, growth hormone-releasing hormone; ANT, antagonist; BDNF, brain derived neurotrophic factor; cAMP, cyclic adenosine monophosphate; CKD, chronic kidney disease; CM, cardiomyocyte; CREB, CRE binding protein; EDR, endothelium-dependent relaxation; HF, heart failure; HFpEF, heart failure with preserved ejection fraction; ISO, isoproterenol; IS, ischemic stroke; LAD, left anterior descending branch; LV, left ventricular; MI, myocardial infarction; MSC, mesenchymal stem cell; NRVMs, Neonatal rat ventricular myocytes; NSC, neural stem cell; OGD, oxygen and glucose deprivation; OPG, osteoprotegerin; ROS, reactive oxygen species; PE, phenylephrine; PKA, protein kinase A; RANKL, receptor activator of NF-*κ*B, ligand; RUNX2, runt-related transcript factor 2; TAC, transverse aortic constriction.

However, the clinical translation of these analogues faces considerable challenges. The primary obstacles include suboptimal pharmacokinetic properties, such as a short half-life, peptide instability, and inadequate blood-brain barrier penetration (Schally et al., 2019). Secondly, the ubiquitous expression of GHRH receptors across tissues raises concerns about long-term safety. The principal risks stem from sustained activation of the GH/IGF-1 axis, which may lead to dose-dependent metabolic disturbances, cardiovascular complications, and requires vigilant neoplastic risk assessment. Potential off-target effects, though less common, and issues like antibody formation also necessitate consideration. Consequently, rigorous monitoring of IGF-1 levels, glucose metabolism, and cardiovascular health is imperative for any long-term therapeutic application. Notably, while *in vitro* studies have raised theoretical concerns about potential proliferative effects on some cancer cell lines, important *in vivo* evidence using MR-409 has demonstrated antitumor activity in xenograft models of various cancers (Schally et al., 2018). This highlights the complexity of translating *in vitro* observations to whole-organism physiology and underscores the need for context-specific evaluation. Additionally, disease heterogeneity, e.g., pathophysiological differences between HFpEF and stroke, the lack of validated biomarkers for patient stratification, the limitation of preclinical data largely to rodent models without validation in large animals, and the need to demonstrate superiority or complementary benefit over existing therapies (e.g., sodium-glucose cotransporter 2 inhibitors) are critical issues that require resolution.

To address these translational challenges, we propose a multifaceted translational strategy and a clear research and development roadmap. A core component is the systematic integration of advanced delivery technologies to overcome the key pharmacokinetic limitations, this includes: 1) Structural optimization using D-amino acid substitutions to enhance stability. 2) Engineered formulations for sustained and targeted delivery, such as biodegradable polymer-based microspheres (e.g., PLGA) for long-term release in chronic cardiovascular conditions, and nanoparticle systems (e.g., surface-modified lipid nanoparticles) or intranasal administration for enhanced brain biodistribution in cerebrovascular diseases (Robles-Fernandez et al., 2025). 3) Developing biased agonists or tissue-homing peptide conjugates to achieve tissue-specific targeting; 4) Prioritizing biomarker development using multi-omics approaches to identify patient populations most likely to respond (Bastos et al., 2025).

This strategy is executed through a phased roadmap. Phase 1 (preclinical optimization) must prioritize the parallel development of analogues and their delivery systems, rigorous efficacy and safety should be validated in clinically relevant large animal models, e.g., aged atherosclerotic minipigs for VC, non-human primate stroke models, and comprehensive preclinical safety assessment, where the choice of delivery platform (e.g., long-acting implant for VC, brain-targeted nanoparticles for stroke) is integral to the study design (Narayan et al., 2025). Phase 2 (clinical development) should adopt a precision medicine framework, stratifying patients using biomarkers. Initial clinical trials on a high-priority indication must include comprehensive pharmacokinetic/pharmacodynamic assessments of the lead formulation, directly comparing delivery efficacy and target engagement to establish a robust foundation for later-phase studies, and employ adaptive phase II trial designs capable of evaluating both cardiovascular and cerebrovascular endpoints simultaneously.

In conclusion, GHRH analogues represent a breakthrough therapeutic strategy, with their ability to target multiple pathological pathways offering new hope for treating complex cardiovascular and ischemic cerebrovascular diseases (Figure 4). However, realizing the potential hinges on systematically addressing translational challenges ranging from pharmacokinetics to long-term safety. Future research must be dedicated to optimizing delivery systems, validating efficacy and safety in advanced models, and executing biomarker-guided intelligent clinical trials. Successfully advancing this roadmap holds the promise of establishing GHRH analogues as a novel class of multi-modal therapeutic agents for tackling currently refractory cardiovascular and cerebrovascular diseases.
